# Coarse-to-Fine Changes of Receptive Fields in Lateral Geniculate Nucleus Have a Transient and a Sustained Component That Depend on Distinct Mechanisms

**DOI:** 10.1371/journal.pone.0024523

**Published:** 2011-09-09

**Authors:** Gaute T. Einevoll, Paulius Jurkus, Paul Heggelund

**Affiliations:** 1 Department of Mathematical Sciences and Technology, Norwegian University of Life Sciences, Aas, Norway; 2 Department of Physiology, Institute of Basic Medical Sciences, University of Oslo, Oslo, Norway; Instituto de Neurociencias de Alicante UMH-CSIC, Spain

## Abstract

Visual processing in the brain seems to provide fast but coarse information before information about fine details. Such dynamics occur also in single neurons at several levels of the visual system. In the dorsal lateral geniculate nucleus (LGN), neurons have a receptive field (RF) with antagonistic center-surround organization, and temporal changes in center-surround organization are generally assumed to be due to a time-lag of the surround activity relative to center activity. Spatial resolution may be measured as the inverse of center size, and in LGN neurons RF-center width changes during static stimulation with durations in the range of normal fixation periods (250–500 ms) between saccadic eye-movements. The RF-center is initially large, but rapidly shrinks during the first ∼100 ms to a rather sustained size. We studied such dynamics in anesthetized cats during presentation (250 ms) of static spots centered on the RF with main focus on the transition from the first transient and highly dynamic component to the second more sustained component. The results suggest that the two components depend on different neuronal mechanisms that operate in parallel and with partial temporal overlap rather than on a continuously changing center-surround balance. Results from mathematical modeling further supported this conclusion. We found that existing models for the spatiotemporal RF of LGN neurons failed to account for our experimental results. The modeling demonstrated that a new model, in which the response is given by a sum of an early transient component and a partially overlapping sustained component, adequately accounts for our experimental data.

## Introduction

Processing in the visual system seems to proceed through processes where coarse information is analyzed before fine details [1], [2]. In striate cortex, single neurons respond with rapid coarse-to-fine changes with respect to several types of stimuli [3]–[9]. Such changes were observed in various experimental conditions including static stimulus presentations with duration similar to typical fixation periods in natural saccadic inspections [6], [10]. Thus, Wörgötter et al. [9] showed rapid shrinkage of subregions in the receptive field (RF) of simple cells during brief (300 ms) static spot stimulation, and consistently Frazor et al. [6] demonstrated increased spatial frequency selectivity during presentations of static (200 ms) grating stimuli. Moreover, in Area V2 of awake fixating macaques, Hegdé and Van Essen [7] showed increasing shape selectivity in single neurons during brief (300 ms) stimulus presentations. The dynamics of such properties have been ascribed to cortical mechanisms [6], [9], [11]. However, several studies have demonstrated significant changes of the spatiotemporal RF also in the dorsal lateral geniculate nucleus (LGN) and retina [12]–[16], and such changes could be an important basis for the coarse-to-fine dynamics at the cortical level. Ruksenas et al. [16] observed transient and rapid shrinkage of the RF-center of LGN-neurons over the first 50–100 ms after onset of a static spot stimulus centered on the RF. Subsequently, the center expanded slightly to a rather stable width that sustained throughout the rest of the stimulus period. Correspondingly, the spatial frequency selectivity of the dLGN neurons increased during static presentations of grating stimuli. The magnitude of these changes was sufficiently large to account for changes observed in striate cortex during related conditions [6], [9].

The mechanisms involved in the coarse-to-fine changes in responses of LGN neurons are unclear, but dependence on a time-lag of the inhibitory surround relative to the excitatory center has been suggested (e.g. [15]). Dynamics of firing rate, which consists of an initial strong and rapidly changing transient component and a subsequent more sustained component (e.g. [17]–[19]), were attributed to a similar lag between center and surround mechanism in both dLGN neurons (e.g. [14]) and retinal ganglion cells (e.g. [20]–[25]). However, rather than simply reflecting a continuous change of balance between an excitatory center and a delayed inhibitory surround, the dynamics of the RF-center width could reflect two distinctly different sets of spatiotemporal mechanisms.

We addressed this question by studying the dynamics of RF-center width of dLGN neurons with particular focus on the transition from the first to the second component. The results indicated that these components reflect two distinctly different spatiotemporal mechanisms that operate with partial temporal overlap. Theoretical analyses demonstrated that existing mathematical models for the spatiotemporal response properties are inadequate for describing these data. We introduce a new model that explicitly treats the response as a sum of a transient and a sustained component. Unlike previous center-surround models, this transient-sustained (TS) model can describe the salient features of our data. This further strengthens the conclusion that the dynamic changes of RF-center size reflect two sets of mechanisms with distinctly different spatiotemporal properties.

## Methods

### Experimental analyses

The experimental methods have been described in detail elsewhere [16]. The procedures were approved by the Norwegian Animal Research Authority in accordance with the Animal Protection Act of Norway. Briefly, adult cats (2.0–3.5 kg) were prepared acutely (arterial and venous cannulation, tracheotomy and craniotomies) under anesthesia induced by xylazine (1.5 mg/kg i.m.) and ketamine hydrochloride (10 mg/kg i.m.), and maintained during surgery by halothane or isofluorane (0.9–1.5%, after induction with 2.5%) in N_2_O/O_2_ (70/30). Local anesthetics (Xylocain; Astra) were applied on pressure points and wound margins. After completion of surgery the animals were immobilized (gallamine triethiodide, initial dose 40 mg, maintenance dose 10 mg/kg/h), and anesthesia was maintained throughout the experiment by halothane or isofluorane (0.4–1.2%) in N_2_O/O_2_ (70/30). EEG was continuously monitored from a pair of silver-wires in left visual cortex (Horsley-Clarke coordinates: posterior 3.5 mm, lateral 2.0 and 10.0 mm). Arterial blood pressure, heart rate, EEG, end tidal CO_2_ (kept at 4%), and rectal temperature (kept at 38°C by a temperature-controlled heating blanket) were also continuously monitored throughout the experiment. Level of anesthesia was adjusted to maintain stable blood pressure, heart rate, and an EEG with dominant frequencies below 4 Hz. To increase the stability of the eyes we made bilateral cervical sympathectomy [26]. We dilated the pupils with atropine, and retracted the nictitating membranes with phenylephrine. The eyes were focused on a video monitor 0.86 or 1.14 m in front of the cat's eyes by means of proper contact lenses.

Extracellular recordings of action potentials from single units in the *A-laminae* of dLGN were made with glass-insulated tungsten electrodes ([27]; exposed tip 6–10 µm), or with glass pipettes filled with 0.9% NaCl (15–25 MΩ *in vivo*). The electrode was inserted perpendicularly through a craniotomy over the left hemisphere at H-C coordinates: anterior 6.0 mm and lateral 9.0 mm. After isolation of action potentials from a single neuron, the RF-center was plotted with hand-held stationary or moving light and dark spots, as well as grating stimuli. The neurons were classified as X or Y, and lagged or nonlagged [19] as described previously [16], [28].

For quantitative studies, we recorded responses to visual stimuli presented on a computer-controlled and gamma corrected, monochromatic video monitor (M21L-0320, Image Systems Corp; phosphor DP104; peak at 565 nm, bandwidth 90 nm; 240 Hz) in front of the cat's eyes. First, the centering and extension of the RF-center was determined with a narrow, flashing slit (bright slits for on-center neurons, dark slits for off-center neurons) presented stepwise across the RF along the horizontal and along the vertical axis. Next, we repetitively presented a series of circular spot stimuli of stepwise increasing diameters centered on the RF. Each spot was presented for 250 ms with a pause of 1000 ms between each spot presentation to avoid sequence effects. Spot size varied from smaller than the RF-center to wider than the whole RF. We presented the spots interleaved such that each spot size was presented once in each series, and such that the whole series of spots was repeated as many times as possible (max 200 times) to achieve best possible spatiotemporal resolution especially in the range of transition between the first and second response component. The spots were luminance increments above (on-center neurons) or decrements (off-center neurons) below a constant, uniform background (0.53 cd/m^2^). Contrast, defined as (L_spot_−L_bkg_)/(L_spot_+L_bkg_), where L_spot_ is spot luminance and L_bkg_ background luminance, was 0.39 for the on-center neurons, and −0.45 for the off-center neurons except for two off-center neurons where it was −0.91; contrasts that gave reasonably balanced peak responses in on- and off-center neurons. We determined the response to each spot size by a peristimulus-time histogram with 5 ms bin width.

To measure temporal changes of RF properties, we made a time-slice through the corresponding bins of all histograms for each 5 ms bin (cf. Fig. 1A in [16]). From the set of response *vs.* spot-width values we obtained for each time-slice, we plotted a spatial summation curve [16], [29]. From this curve, we estimated three RF parameters. First, we estimated center size by the width of the spot that elicited maximum response. Second, surround width was estimated by the width of the spot just large enough to give minimum response. Third, to estimate center-surround antagonism we determined the difference of response to the spot that just filled the center and the one that just filled both center and surround. We defined center-surround antagonism as the ratio between this difference and the center response [16], [29], [30]. The dynamics of the RF-properties were determined from changes of the respective estimates throughout the series of time slices.

We carefully monitored the data-acquisition during the experiments to avoid distortion of results due to shifts in eye-position. By possible indications of shifted eye-position, we stopped data-acquisition and checked the centering of the stimulus on the RF. If necessary, we corrected the centering, discarded the collected data, and restarted the data-acquisition. After completed acquisition of the spatial summation data, we repeated the determination of the centering of the RF with flashing slits along the horizontal and vertical axis to control for possible shifts in eye-position. To reduce the risk of error of measurement due to undetected eye-movements, we preferentially sampled neurons with RF outside *area centralis*. We always kept the non-dominant eye covered during recordings.

At the end of the experiment, the animal was deeply anesthetized with pentobarbitone sodium (50 mg/kg i.v.) and perfused transcardially with saline followed by 4% formaldehyde in saline. We verified electrode positioning histologically from Nissl-stained brain sections.

### Mathematical modeling

Several mathematical models for the spatiotemporal response *R*(*t_i_,d_j_*) were considered, and to assess model performance a least-squares *relative error* measure was used, i.e.,
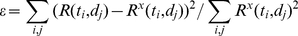
(1)where *R^x^*(*t_i_,d_j_*) is the experimental data. Further, *i = 1,…,N_t_* and *j = 1,…,N_d_* where *N_t_ = 49* is the number of time bins, and *N_d_* is the number of different spot diameters used. For the time-resolved fits to difference-of-Gaussians (DOG) functions, cf. Eq. (4) below, we also used the *time-resolved relative error*

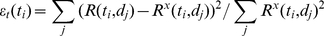
(2)In the *principal components analysis (PCA)* the data are expanded in terms of principal components as described by Gershenfeld [31],
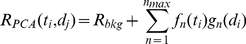
(3)where *n = 1,…,n_max_* is the number labeling the principal component, and *n_max_* is the total number of principal components included in the analysis. The background firing rate *R_bkg_* was found by averaging the background response occurring prior to the first stimulus-evoked response.

In the *time-resolved DOG fits* the DOG formula [32] was fitted against area-summation curves for each time slice separately. Formally, this time-resolved DOG-model is given by,
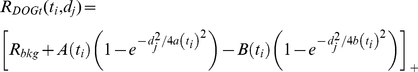
(4)where *A*(*t_i_*), *B*(*t_i_*), *a*(*t_i_*) and *b*(*t_i_*) (*i = 1,…,N_t_*) are parameters to be fitted, and [*x*]_+_ is the half-wave rectifying function (0 for negative *x*, *x* for positive *x*) assuring non-negative model firing rates [33]. The MATLAB routine *fminsearch* was used in the optimization, i.e., to minimize the least-squares relative error *ε_t_(t_i_)* in Eq. (2) for each time step separately. We also did time-resolved fits to a pair of DOG functions. This time-resolved 2-DOG-model is given by
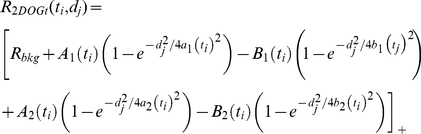
(5)


The *center-surround models* are given by,
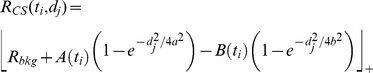
(6)where the choices of functional forms of *A*(*t_i_*) and *B*(*t_i_*) may vary [14], [33]. For example, Freeman and colleagues [14], [15] used a specific form where the time derivatives of the functions *A*(*t*) and *B*(*t*) are essentially of the form,
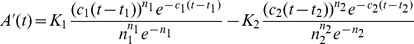
(7)where *B′*(*t*)* = A′*(*t−t_d_*). In the present fitting to data we instead determine the parameters *A*(*t_i_*) and *B*(*t_i_*) (*i = 1,…,N_t_*) *non-parametrically* using (i) techniques from linear estimation to estimate best fits of *A*(*t_i_*) and *B*(*t_i_*) given choices for the model parameters *a* and *b*, and (ii) the MATLAB routine *fminsearch* to find values of *a* and *b* giving the overall lowest error *ε*. With 49 time bins and 2 parameters (*A*,*B*) to fit for each time bin (*R_bkg_* was found by averaging the response for the earliest time bins) plus the two width parameters *a* and *b*, this gave a total of 100 fit parameters.

In the new *transient-sustained (TS) model* the response is modeled as a sum over a transient (*R_t_*(*t,d*)) and a sustained part (*R_s_*(*t,d*)), i.e.,
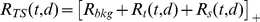
(8)The *transient* part is modeled as a sum over two functions consisting of DOGs multiplied with different temporal functions, i.e.,
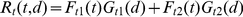
(9)Here the DOG functions are given by [34],
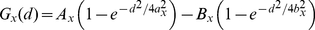
(10)where the subscript *x* represents *t1* or *t2*. The first temporal function *F_t1_* is modeled as the (integrand of the) Gamma function [14], i.e.,
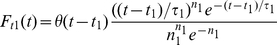
(11)where *θ(t)* is the unit step function. The second temporal function *F_t1_* is essentially modeled as the derivative of this function, i.e.,
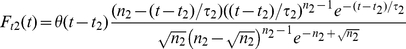
(12)Both *F_t1_*(*t*) and *F_t2_*(*t*) are normalized such that their maximum values are one.

The *sustained* part is modeled as a DOG with an exponential onset, i.e., *R_s_*(*t,d*)* = F_s_*(*t*)*G_s_*(*d*). Here *G_s_*(*d*) is of the form in Eq. (10), and
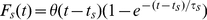
(13)where *t_s_* and *τ_s_* are the onset time and time constant of the sustained component, respectively. The complete TS-model applicable for *non-lagged cells* thus reads,
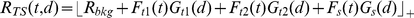
(14)


In the fits to experimental data for the non-lagged cells, the parameters *A_s_*, *B_s_*, *a_s_*, *b_s_* for the DOG function *G_s_*(*d*) of the sustained part are first fitted to the last part of the data, i.e., the data 125 ms or more after spot onset. Then the parameters describing *F_s_*(*t*), *F_t1_*(*t*), *F_t2_*(*t*), *G_t1_*(*d*), and *G_t2_*(*d*), are determined in an overall fit against the experimental data using MATLAB's *fminsearch* routine. The coefficients *n_1_* and *n_2_* in the functions *F_t1_*(*t*) and *F_t2_*(*t*), respectively, were constrained to be less than 15. In the numerical fitting all model parameters except *R_bkg_* and the time of onset of the sustained part (*t_s_*) were varied, leaving a total of 19 model parameters to fit.

For *lagged cells* a simplified model was chosen where the transient components are omitted and only the sustained component remains, i.e.,
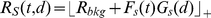
(15)


The spatiotemporal impulse-response function *D_TS_*(*t,r*) [33] for the TS-model in Eq. (14) is given by
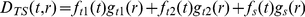
(16)where the spatial functions *g_m_*(*r*) (*m = t1,t2,s*) are DOG functions
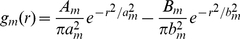
(17)and the temporal functions *f_m_*(*t*) are found from temporal differentiation of the temporal response functions *F_m_*(*t*) in Eqs.(11–13), i.e., *f_m_*(*t*)* = dF_m_*(*t*)*/dt*. This gives
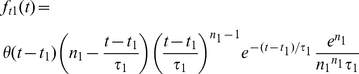
(18)

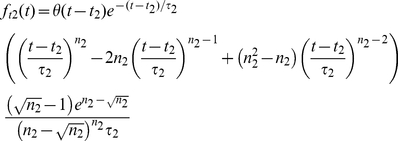
(19)and
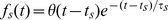
(20)To facilitate comparison with previous results [14], [15] we also give the expression of the ‘one-dimensional impulse response’, i.e., the response to thin vertical bars. This impulse-response function is also of the form given in Eq. (16), but with the spatial functions *g_m_*(*r*) replaced by a new function *g_bar,m_*(*x*) (found by straightforward spatial integration of *g_m_*(*r*) in the *y*-direction):
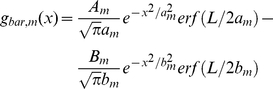
(21)This expression applies for a thin bar (bar width much smaller than *a_m_* and *b_m_*) and length *L* positioned perpendicularly to and symmetrically around the *x*-axis. The function *erf*(*x*) is the so called *error function*.

## Results

### Experimental analyses

We studied neurons from *A-laminae* of LGN with RFs within 30 deg from *area centralis* (N = 51; 32 X-, 19 Y-neurons; 14 X-neurons were lagged). There was no overlap between this set of neurons and the set of neurons in our previous study [16]. For each neuron, we recorded responses to presentation (250 ms) of a series of spots (light spots for on- and dark spots for off-center neurons) centered on the RF. Spot width was stepwise increased from considerably smaller than the RF-center to larger than the whole RF. Temporal RF-changes during spot presentation were analyzed based on time-slicing across peri-stimulus-time histograms for the response to the series of spots (cf. Fig. 1 in [16]). We estimated the RF-parameters at a given time from a spatial summation curve across the respective time-slice; e.g., we determined the width of the RF-center by the diameter of the spot that elicited maximal response on the assumption that this spot just covered the RF-center. Since a major purpose of this series of experiments was to obtain detailed insight into the spatiotemporal RF, particularly concerning the changes in the interval of transition between the primary transient response and the secondary sustained response, we repeated the presentation of the spot series as many times as possible to achieve adequate spatiotemporal resolution.

#### Changes of RF-center size: two components with different spatiotemporal properties

**Figure 1:**
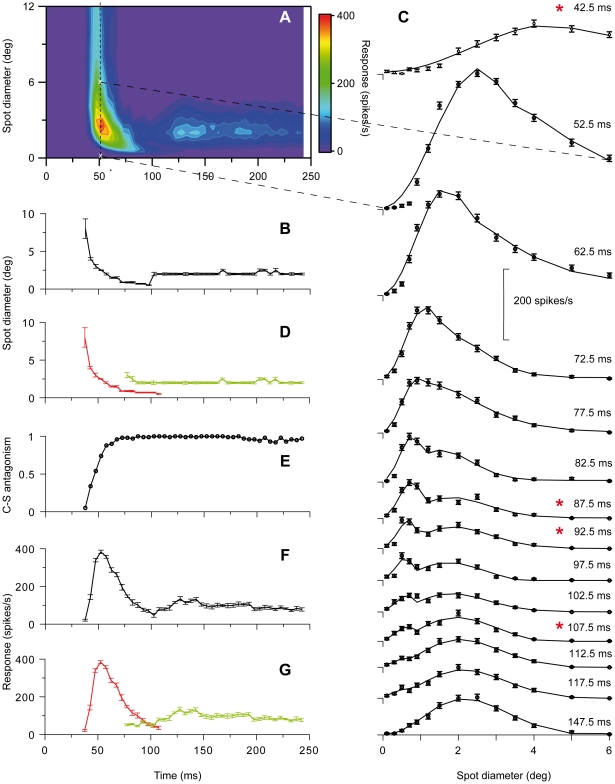
RF dynamically changes during brief stimulus presentation. Data from an on-center Y-neuron. ***A***, Colormap image of response (z-axis) to a series of spots (n = 25) of different diameters (y-axis) at different time after spot onset (x-axis). Spots were centered on the RF. ***B***, Center-width as function of time after spot onset. Center-width was determined by spot diameter giving maximal response. The RF-center shrank from initially 8 deg to a minimum of 0.5 deg and then increased to a stable width of 2 deg at ∼100 ms. ***C***: Spot width tuning curves for a selected number of time-slices. Notice the truncated x-axis. The time-slice for the spatial summation curve at 52.5 ms is marked by the vertical dashed line in (A), and the first and last data point in this curve are marked by white crosses in (A). Notice the shoulder or bimodal appearance of the curves in the range of 72.5 and 107.5 ms. Single (Eq. 4) and double (Eq. 5) DOG-functions were fitted to the data. Continuous curves show the best-fitting 2-DOG function (linearly interpolated between the spot sizes corresponding to experimental data points). Cases in which the 2-DOG gave statistically better fit than the best-fitting single DOG are marked with asterisk. ***D***, Replot of data in (B) where center width of the transient (red curve) and sustained component (green curve) are separated based on the estimated start of the sustained component, and the end of the first component. ***E***, Development of center-surround antagonism. Notice that 100% antagonism was reached within the first 70 ms. ***F***, Development of the firing rate to the spot that just filled the RF-center. ***G***, Data from (F) separated for the transient (red) and sustained (green) components. Error bars are ±SE. Number of presentations of each spot, 200.

We found pronounced changes of RF-center width during spot presentation consistent with our previous study [16]. The changes consisted of an initial transient component characterized by rapid shrinkage of the RF-center followed by a second component characterized by an initial minor center expansion to a subsequent relatively stable size. This is illustrated in Fig. 1 by results from a representative on-center nonlagged Y-neuron. Fig. 1A shows a color-map image of the response (z-axis) to the set of spot width (y-axis) plotted against time after spot onset (x-axis). Notice the increasing latency to peak response during the first response component, that is, the curved shape of the color map in the bottom left corner. Due to low firing rate at the start of the visual response, we determined the initial spatial summation curve for the first time-slice where the maximal visual response was at least twice the average spontaneous activity. Thus, the timing of the first time-slice does not express the very start of the visual response.

**Figure 2:**
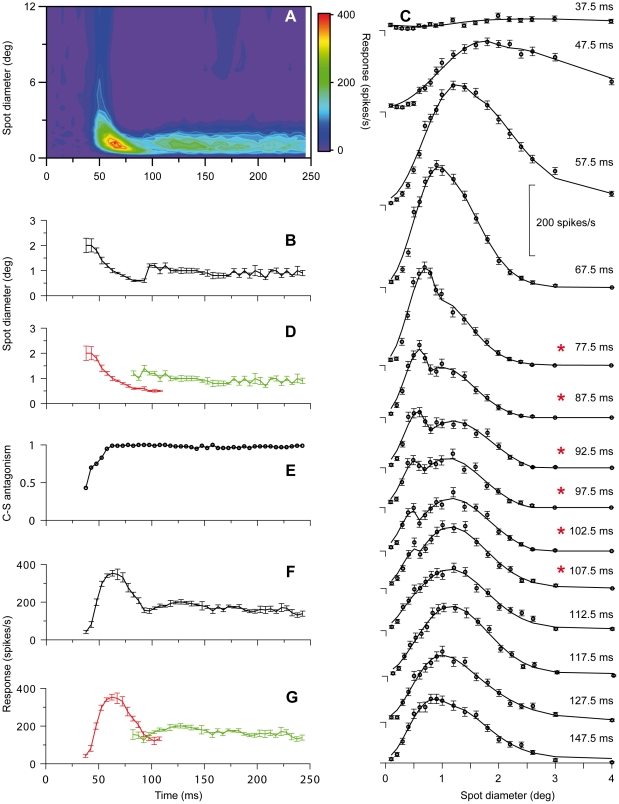
RF dynamics for an on-center X-neuron. Similar plots as for the Y-neuron in Fig. 1. Number of presentations of each spot 125.

Interestingly, the color-map image suggests that there is a discontinuity rather than a continuous change at the transition between the dynamic initial response and the later more sustained component. The possible discontinuity is even more apparent in Fig. 1B where RF-center diameter is plotted against time after spot onset. Such discontinuity could indicate that the dynamic change of the RF during the 250 ms stimulus period involved two distinctly different sets of neural mechanisms. Figs. 2A and 2B illustrate similar results for a representative on-center X-neuron.

**Figure 3:**
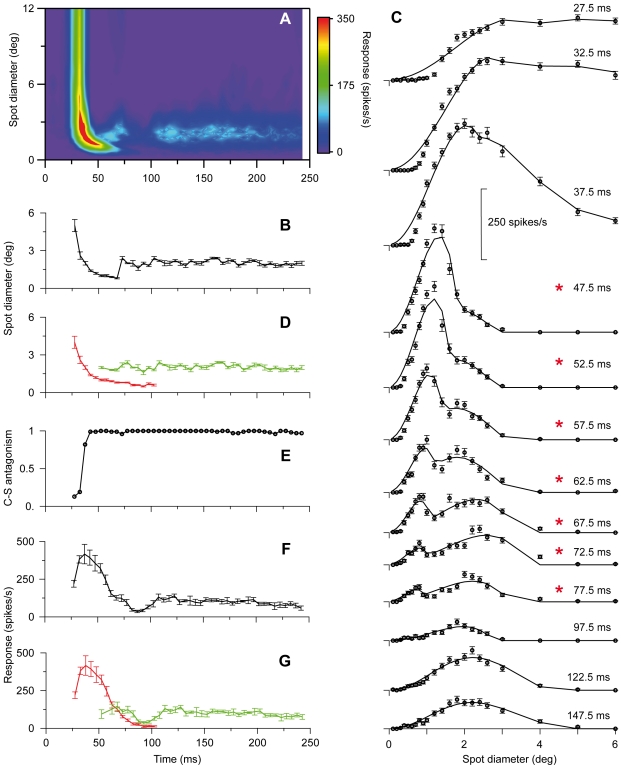
RF dynamics of an off-center Y-neuron. Similar plots as for the Y-neuron in Fig. 1. Number of presentations of each spot 115.

The initial rapid response changes for the neurons illustrated in Figs. 1 and 2 could partly be due to fast luminance adaptation since the spot stimulus for on-center neurons was a luminance increment above the constantly presented background of fixed luminance. This is unlikely because similar changes occurred for off-center neurons for which the spot stimulus was a luminance decrement below the background luminance as illustrated in Fig. 3 by results for a representative off-center Y-neuron.

The initial shrinkage of the RF-center occurred in all nonlagged neurons (n = 37). On average, the initial field center was 4.5±2.9 (SD) times wider (p<0.001, paired t-test) than the minimum center width. The center subsequently widened to on average 2.2±1.2 times minimum center width (n = 37). These values are consistent with our previous results [16]. The degree of shrinkage during the transient component was more pronounced in Y- than in X-neurons; initial width was 6.0±3.0 times minimum width for Y-neurons (n = 19), and 2.8±1.7 for the non-lagged X-neurons (n = 18; p<0.001). The mean increase from minimum width to the average width during the sustained component was also larger for Y-neurons (2.7±1.4 times) than for X-neurons (1.7±0.6 times; p<0.001).

In most time-slices, it was difficult to determine a reasonably precise estimate of the width of the RF-surround due to the low rate of response change to the wide spots. Nevertheless, it was possible in most cases to estimate center-surround antagonism. The strongest antagonism we could determine, termed 100%, occurred when the surround inhibition became sufficiently strong to prevent firing of action potentials. In most neurons, the center-surround antagonism increased rapidly from weak to 100% antagonism during the transient component (Figs. 1E, 2E, 3E).

#### Partial temporal overlap of the two components indicates contributions from two distinct sets of neural mechanisms

Detailed analyses of differences between the spatial summation curves at different time-slices provided strong evidence for two distinct spatiotemporal components. The spatial summation curves in the beginning of the series of time-slices were unimodal, and through the successive curves, the peak shifted toward smaller spot sizes reflecting the shrinking RF-center of the transient component. However, in the interval of transition between the two components, an inflection or shoulder in the falling part of the spatial summation curves occurred, and in the subsequent time-slices, this shoulder could develop into a local maximum giving the curves a bimodal shape (Figs. 1C, 2C, 3C). This shoulder or second peak occurred at spot widths corresponding to the center width of the sustained component indicating parallel and simultaneous generation of the sustained and the transient component in this transition interval. During the successive time-slices in the transition interval, the peak related to the transient component continued to shift toward smaller spot widths while the amplitude gradually decreased until the peak eventually disappeared ∼120–130 ms after stimulus onset (Figs. 1C, 2C, 3C). Meanwhile, the amplitude of the peak related to the sustained component was relatively stable such that the spatial summation curves eventually regained a unimodal shape (Figs. 1C, 2C, 3C).

This complex shape of the spatial summation curves in the transition between the transient and sustained response components was noticed in all non-lagged neurons except for two X-neurons. The neurons with the largest shrinkage of RF-center tended to have the most pronounced bimodal shape of the curves, whereas in neurons with smaller degree of shrinkage the peaks related to the two response components were less clearly separated. Accordingly, Y-neurons had more pronounced bimodal shape of the summation curves in the transition interval than X-neurons. In the two deviating X-neurons we could not exclude the possibility that the steps in spot sizes, used in the series of stimuli to detect a possible shoulder or double peak, were too large.

The characteristics of the spatial summation curves in the transition interval between the transient and the sustained component indicate involvement of two distinctly different sets of neuronal mechanisms that contribute simultaneously to the response in this interval. Clearly, the transient component was generated from a source with strong RF-center dynamics, and the sustained component from a source with more stable RF-center size. Both sources have antagonistic center-surround organization as demonstrated in the spatial summation curves by the gradual response reduction as the spot widths became increasingly wider than the putative RF-center. Moreover, the center-surround antagonism of the transient component had a pronounced development from little or none antagonism at the start of the response, to a very strong one. Possible temporal changes of center-surround antagonism in the RF for the sustained component during the spot stimulation was difficult to determine, but clearly, they were small compared to those of the transient component.

To further investigate the hypothesis that the changes of RF-center width reflect contributions from two distinct sets of mechanisms that both have antagonistic center-surround organization, we fitted two different mathematical functions to the set of spatial summation data in each time slice (cf. Methods). One of the functions is based on the assumption that the data reflected a single DOG function (Eq. 4), the other that the data reflected a sum of two DOG functions with different spatial and temporal characteristics (Eq. 5). The rationale for choosing the 2-DOG function is that it represents a natural extension to the single-DOG function, and can simply account for response curves with two maxima. The results showed that this 2-DOG function did not give a significantly better fit than a single DOG function to data in early time slices during the transient component, or to the data in the late time slices during the sustained component. However, for data in time-slices in the transition between the two components, the 2-DOG function gave a significantly better fit than the single DOG function (p<0.05, F-test) for 21 of the 35 neurons. The remaining neurons showed less pronounced separation of the two components, and the inflections in the transition region between the transient and sustained part of the response was most likely not large enough to give a statistically significant difference between the best fit of the two DOG functions. In Figs. 1C, 2C, 3C the continuous curve shows the best-fitting 2-DOG function, and time slices marked by an asterisk show cases in which the 2-DOG function gave a significantly better fit than the single DOG function.

The fit of the 2-DOG-functions to the spatial summation curves showed an interesting systematic deviation for the response to the smallest spots during the transient response components. For this range of spots, the best-fitting curves had a smaller rate of change than indicated by the data points (Fig. 1C, 42.5–97.5 ms; Fig. 2C, 47.5–77.5 ms; Fig. 3C, 27.5–67.5 ms). Thus, at small spot sizes the response vs. spot-width increased at a higher rate during the transient response component than accounted for by the 2-DOG functions, indicating that the model is inaccurate in this region.

With our method, the start of the sustained response component and thereby the start of the interval over which the transient and sustained components occurred simultaneously, was detected by the inflection in the falling part of the spatial summation curve (e.g. Fig. 1C, 82.5 ms). However, the real start must have occurred even earlier. As a putatively conservative estimate of the start of the sustained component, we took the time of the last time-slice in the beginning of the series at which no inflection in the falling part of the spatial summation curve was noticeable. This estimated start varied in the sample of neurons between 45 and 90 ms after stimulus onset with a mean of 62.5±12 ms (N = 35). There was no statistically significant difference between X- and Y-neurons. The last appearance of the transient component, and thereby the last simultaneous appearance of the two components, was noticeable by a minor notch near the start of the rising part in the spatial summation curve (e.g. Fig. 1C, 112.5 ms). To estimate the end of the transient component we took the time of the first time-slice after the disappearance of this notch. Thus, the estimated disappearance of the transient component varied in the sample of neurons between 95–145 ms after stimulus onset with a mean of 123±13 ms. Also for this quantity there was no statistically significant difference between X- and Y-neurons. The average length of the interval of overlap according to these estimates was 59±18 ms. Based on the estimated start of the second component and the end of the first component, we replotted center width against time (Figs. 1D, 2D, 3D) to demonstrate the overlap between the two components (transient in red, sustained in green).

To control for the possibility that the shoulder or bimodal shape of the summation curves in the transition between the transient and sustained component could be due to a shift of eye-position during the recordings, we verified that the centering of the RF was the same before and after the experiments on spatial summation (cf. Methods). Moreover, the gradual shift of the peak related to the transient component combined with the relatively stable position of the peak to the sustained component is inconsistent with the hypothesis that the inflection or bimodality was due to a shift of eye-position during the recording. Furthermore, for neurons for which we had sufficiently strong response, we compared the spatial summation properties determined from the response to the first fifty presentations of the series of spots with the properties determined from the last fifty presentations, and showed that the characteristics were similar in the two cases.

#### Lagged neurons lack the initial shrinkage of the RF-center

**Figure 4:**
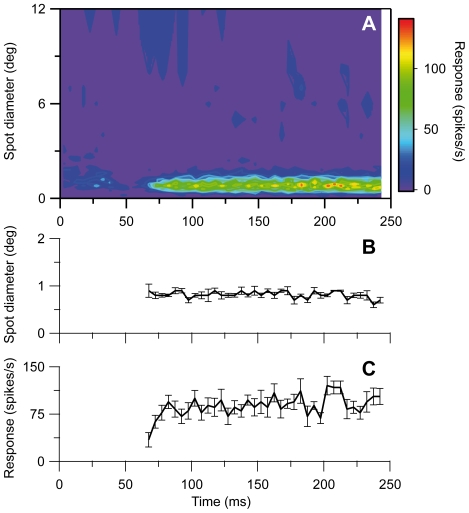
RF dynamics of a lagged on-center X-neuron. Similar plots as for the Y-neuron in Fig. 1. In (A), notice the initial suppression of the response. Number of presentations of each spot 70.

The lagged neurons did not show any marked change of RF-center width during the spot stimulation period [16], also not with the increased spatiotemporal resolution of the method used in the present series of experiments. On the contrary, the center width remained remarkably stable during the period of visual response (Fig. 4). Notice that instead of the pronounced initial shrinkage seen in nonlagged neurons, the lagged neurons are initially suppressed during spot stimulation [19], [35], [36].

#### Relation to the transient and sustained components of firing rate

The dynamic changes of RF-center width in nonlagged neurons occurred in parallel with the well-known dynamic changes of firing rate during spot presentation (e.g. [17]–[19]). The rapid initial shrinkage of the RF-center occurred during the initial transient firing (compare Figs. 1B and 1F, 2B and 2F, and 3B and 3F), but the relationship between center width and firing rate was not monotonic. During the interval when the RF-center gradually shrank, the firing rate increased to a maximum (at ∼60 ms in Fig. 1F), where after it rapidly decreased. However, center width and firing rate had similar timing in the sense that both properties had an initial dynamic component and a later largely sustained component. Moreover, the dynamic component in both cases occurred within the same time interval, suggesting a common underlying dynamic mechanism. Correspondingly, the sustained component occurred within the same time interval with respect to both firing rate and RF-center width.

The firing rate usually had a secondary peak at the beginning of the sustained firing component, but we observed no consistent monotonous relationship between these changes and possible changes of center width in our data.

### Mathematical modeling

#### Comparison with existing spatiotemporal receptive-field models

**Figure 5:**
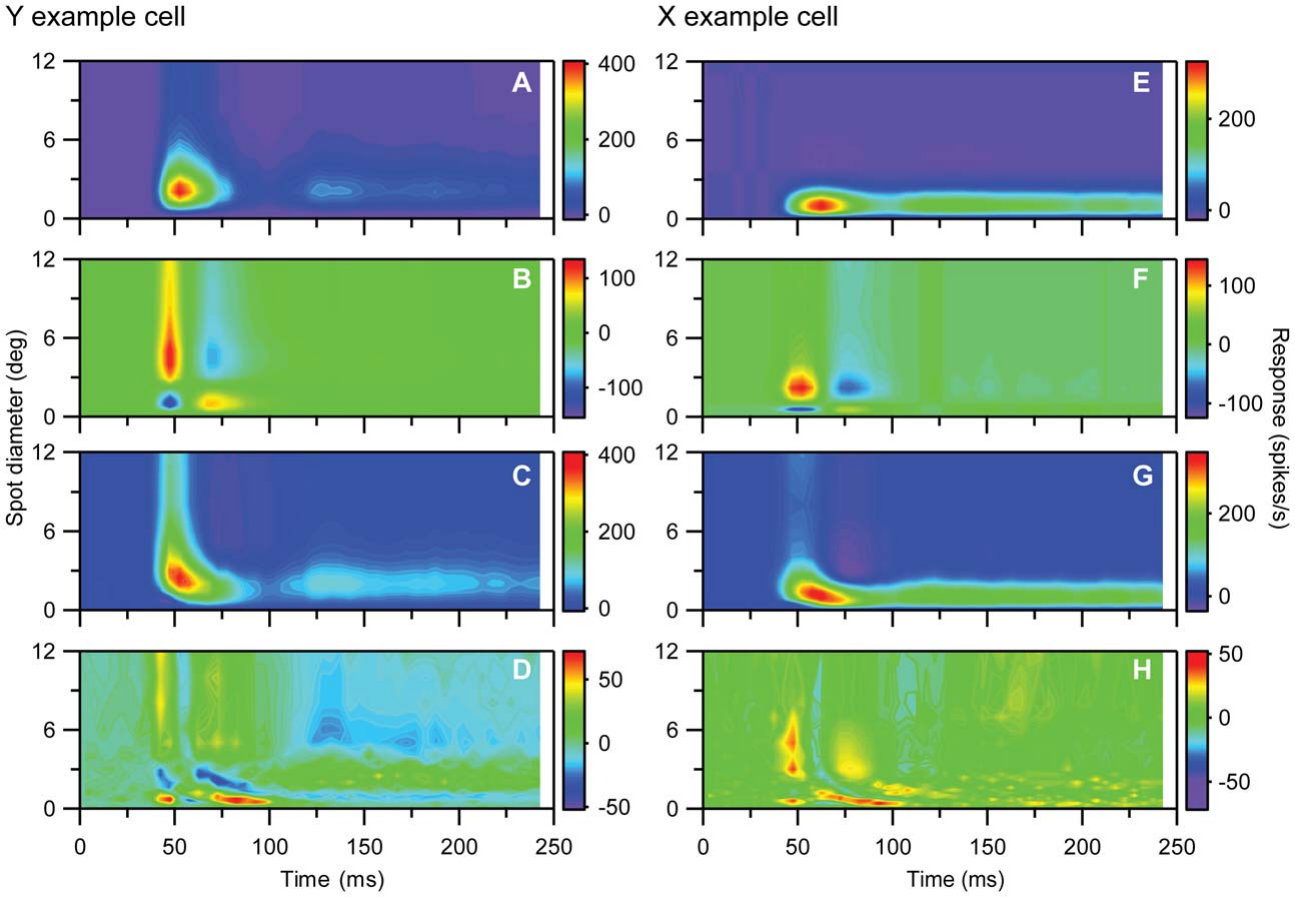
Principal components analysis (PCA) for example on-center Y and X neurons in **Figs. 1** and **2**. ***A***, ***B***, 1^st^ and 2^nd^ principal components, respectively, for the Y-neuron response data, i.e., contributions from terms with *n = 1* and *n = 2* in Eq. (3). ***C***, Sum of contributions from two first principal components (and background activity) for Y neuron. ***D***, Deviation between experimental results for Y neuron and PCA results in (*C*). Error *ε* (cf. Eq. 1) is 0.036. ***E–H***, Same as (*A*)–(*D*) for the X-neuron response data. The deviation between experimental results and PCA results (*G*) corresponds to an error *ε* = 0.015.

We next investigated to what extent various existing mathematical models for the spatiotemporal response can account for the experimental data. We first performed a *principal components analysis* (*PCA;* [31]) to get insight into the level of model complexity needed to account for the data. The modeling was based on the results in Figs. 1 and 2 which were representative for the basic response properties of the Y- and X-neurons, respectively. With PCA the experimental spatiotemporal response data was expanded into a sum over spatiotemporally separable components (Eq. 3) where the first component accounts for as much of the data as possible, the second component for as much as possible of the data unaccounted for by the first component, and so on. For the Y and X example neurons depicted in Figs. 1 and 2, respectively, these two first components together account for 96% or more of the stimulus-evoked data: the error *ε* (Eq. 1) by including two components in the sum in Eq. (3) is found to be 0.036 and 0.015 for the Y- and X-neurons, respectively. The resulting two first principal components for these example Y- and X-neurons are shown in Fig. 5. This figure illustrates further that the shrinking of RF-center width is captured well by the sum of the two first principal components, while the first principal components alone are insufficient. The latter observation is as expected since keeping only the first principal component amounts to assuming a model expression for the stimulus-evoked activity of the form *R*(*d,t*)*−R_bkg_ = f_1_*(*t*)*g_1_*(*d*). With such a spatiotemporally separable response function, the RF-center size will by necessity be constant over time since it is only determined by the function *g_1_*(*d*).

**Figure 6:**
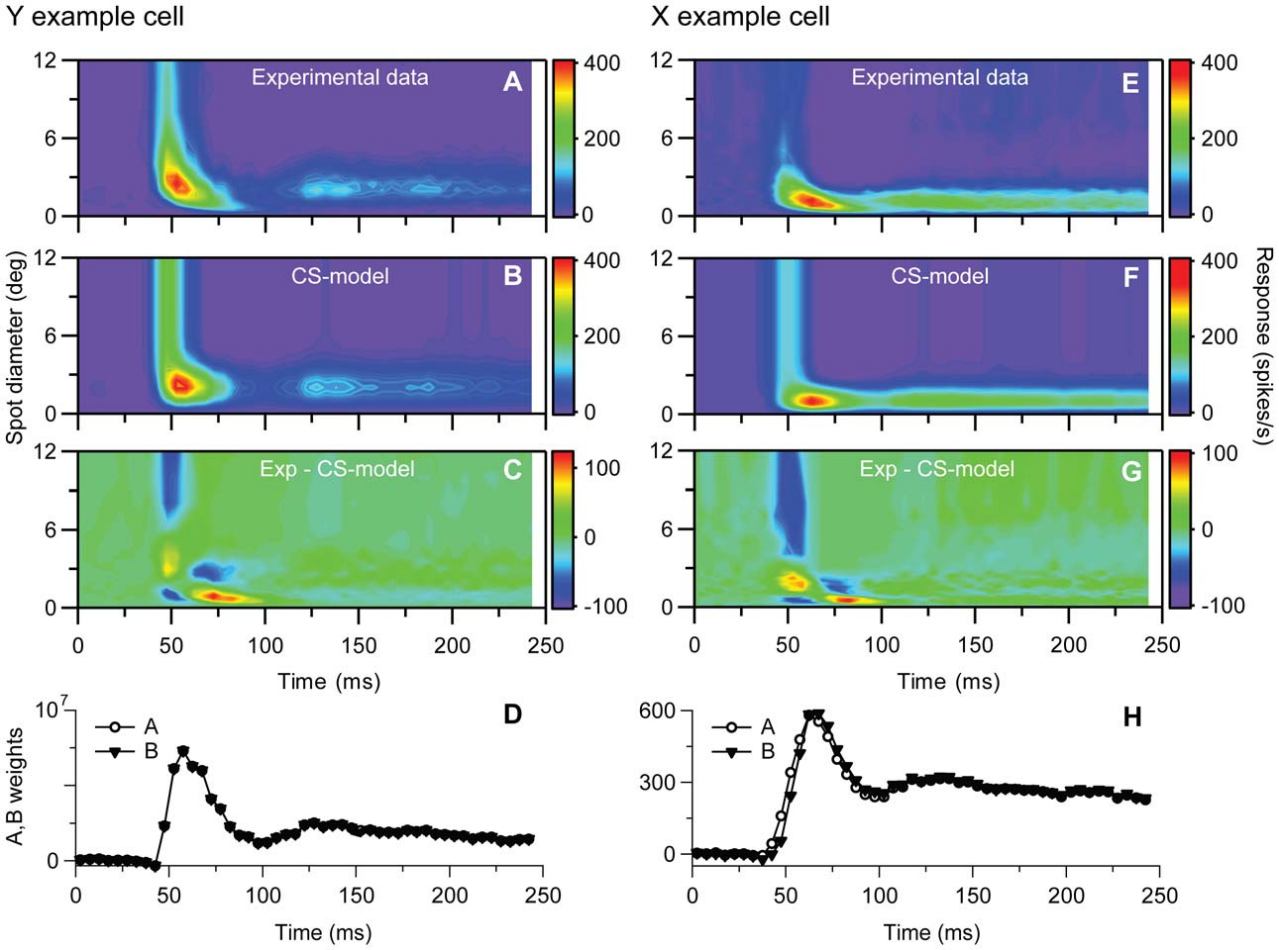
Fits to center-surround (CS) model for example on-center Y and X neurons in **Figs. 1** and **2**. ***A***, Experimental Y-neuron response data. ***B***, Best fit to CS-model in Eq. (6) with non-parametric representation of *A*(*t_i_*) and *B*(*t_i_*). ***C***, Deviation between experimental results (A) and CS model results in (B). Error *ε* (cf. Eq. 1) is 0.071. ***D***, Fitted values of weight parameters *A*(*t_i_*), and *B*(*t_i_*), cf. Eq. (6), ***E–H***, Same as (A)–(D) for the X-neuron response data. The deviation between experimental results (*E*) and CS model results (*F*) corresponds to an error *ε* = 0.040.

The conclusion from this PCA analysis is that if we stick to models based on sums of spatiotemporally separable component functions, at least two separate components are needed. An example of such a two-component model is the *center-surround model* (Eq. 6) which has been used previously to describe spatiotemporal response properties of dLGN neurons [14], [15]. In these applications, specific choices for the temporal functions *A*(*t*) and *B*(*t*) in Eq. (6) were made (cf. Eq. 7). Here we are less restrictive and allow for non-parametric fits of *A*(*t*) and *B*(*t*), which means that the values *A*(*t_i_*) and *B*(*t*_i_) are allowed to vary freely for each time bin *t_i_*. The best fits of the center-surround model with non-parametric time-dependent weights (Eq. 6) to the experimental responses for the example Y and X cells are shown in Fig. 6. For both examples we observe that the model cannot reproduce the salient RF shrinking effect for short times. The fitting errors, *ε*, were found to be 7.1% and 4.0%, respectively. Any model of the CS-type in Eq. (6) where the center (*A*(*t_i_*)) and surround (*B*(*t_i_*)) weights have *specific* functional forms [14], [15], [33], will by necessity have less flexibility than this non-parametric model, and should give even poorer fits.

While center-surround models of the form in Eq. (6) with fixed center and surround widths were found to be inadequate for describing the RF shrinking effect, the fitted values of the center (*A*(*t_i_*)) and surround weights (*B*(*t_i_*)) for the center-surround model from the X-neuron fit in Fig. 6 were found to be in qualitative accordance with results from previous studies: in Fig. 6H the surround weight *B*(*t_i_*) is seen to be similar to, but lag the center weight *A*(*t_i_*) with a few milliseconds for this X-neuron, in accordance with previous observations [14], [15]. Further analysis of the time-derivatives of the fitted weights *A*(*t_i_*) and *B*(*t_i_*) for this X-neuron also revealed that they could be well fitted by the functions suggested in Cai et al. [14], cf. Eq. (7) (results not shown). For the example Y-neuron, however, no such systematic lag between the center and surround weights was found. As seen in Fig. 6D the fitted center (*A*(*t_i_*)) and surround weights (*B*(*t_i_*)) are both extremely large and essentially identical with each other for all time slices for this neuron. However, since the fitted center width *a* is only slightly different from the fitted surround width *b*, the two huge center and surround contributions almost cancel each other completely, leaving only a (relatively speaking) small net model response. These unphysiologically large center and surround components in the best fit further point to the inadequacy of the CS-model in accounting for the example Y-cell data.

#### Fits to time-resolved DOG models

**Figure 7:**
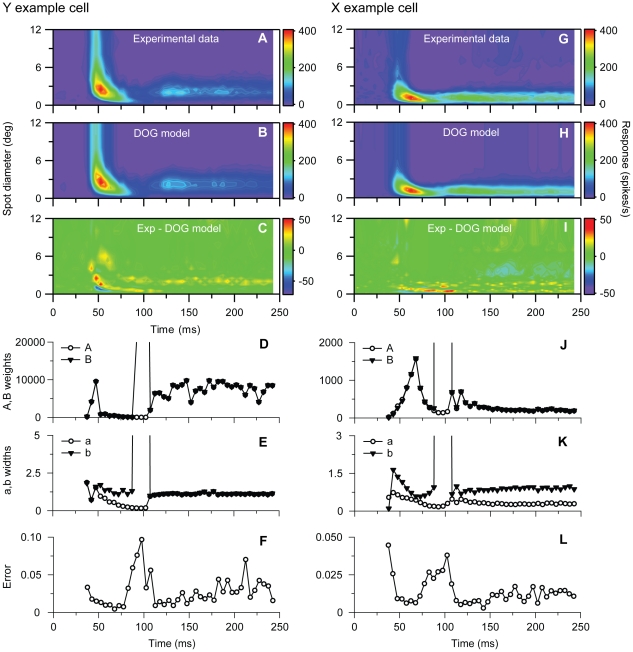
Fits to time-resolved DOG functions for example on-center Y and X neurons in **Figs. 1** and **2**. **A**, Experimental Y-neuron response data. **B**, Best fit to time-resolved DOG model in Eq. (4). **C**, Deviation between experimental results (A) and model results in (B). Error *ε* (cf. Eq. 1) is 0.016. **D**, Fitted values of weight parameters *A*(*t_i_*) and *B*(*t_i_*) for Y neuron. **E**, Fitted values of width parameters *a*(*t_i_*) and *b*(*t_i_*) for Y neuron. **F**, Time-resolved error *ε_t_* (cf. Eq. 2) for Y neuron. **G–L**, Same as (A)–(F) for the X-neuron response data. The deviation between experimental results (G) and model results (H) corresponds to an error *ε* = 0.012. Note that the almost vertical lines in panels (D) and (J) signal a rapid growth of the fitted value of the weight parameter *B* to values beyond the maximum values of the y-axes. The almost vertical lines in panels (E) and (K) correspondingly signal a rapid growth of the width parameter *b*.

The clear conclusion from the above fits is that any center-surround model of the type in Eq. (6), where the spatial widths of the center and surround terms are fixed to a constant value, is incapable of accounting for the present data and in particular the salient features of the time-dependence of the RF-center sizes. We thus needed to search for other model types. To help elucidate the form such a new model must have, we next fitted the standard *difference-of-Gaussians (DOG)* model to time-resolved response data, cf. Eq. (4). As can be seen in Fig. 7 such a set of DOG models is in general able to account well for the salient features of the response data for the example Y- and X-neurons. The total errors *ε* of these best fits are 1.6% and 1.2%, respectively, and the shrinkage of the RF-centers at short times is well captured. This is not surprising since a large number of model parameters are allowed to vary freely, 4 parameters for each time bin multiplied by the number of time bins which here is 49.

In Figs. 7D, 7E, 7J, 7K we show the time dependence of these fitted DOG parameters for our example cells. In Figs. 7D and 7J we see that the fitted center (*A*(*t_i_*)) and surround weights (*B*(*t_i_*)) mostly follow each other closely and are very similar, even though both are strongly time dependent. Note that the fitted surround weights (*B*(*t_i_*)) become very large for some time bins around 100 ms and are beyond the maximum values of the depicted axes.

Unlike the weights, the fitted center (*a*(*t_i_*)) and surround. widths (*b*(*t_i_*)) are seen to have very different time dependencies. An exception is the times beyond about 110 ms for the Y-cell where the center width is only slightly smaller than the surround width so that the shapes of the center and surround contributions are almost identical.

In Figs. 7F and 7L we finally show the time dependence of the relative fitting error *ε_t_*, Eq. (2), for the example Y- and X-neurons. The relative fitting error is seen to be at a maximum at around 100 ms indicating that the response data is poorly described by a single DOG function at these times. This further hints that more than one mechanism is evoked and overlap at these times.

The time-variation of the widths are seen in Figs. 7E and 7K to be particularly large for times less than ∼110 ms after stimulus onset. This observed time-dependence hints at why *center-surround* models of the type in Eq. (6), where center and surround terms with fixed spatial widths and time-dependent weights, are not well suited to account for the present data. If anything, a new type of center-surround model with equal center and surround weights, but time-dependent (and different) spatial widths, is suggested, i.e.,
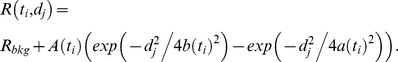



#### Transient-sustained (TS) model

The direct observations of two separate components with different spatiotemporal properties in the experimental data in Figs. 1, 2, 3 combined with the observation in Fig. 7 that a single DOG is insufficient to account for spatial responses for times around 100 ms after stimulus onset, suggest a new two-component model, the *transient-sustained (TS)* model, cf. Eq. (8). In this TS model the response is given as a sum over two components: an early *transient* component (*R_t_*(*t,d*)) lasting up to about 120 ms after stimulus onset, and a partially overlapping *sustained* component (*R_s_*(*t,d*)) starting about 60 ms after onset.

**Figure 8:**
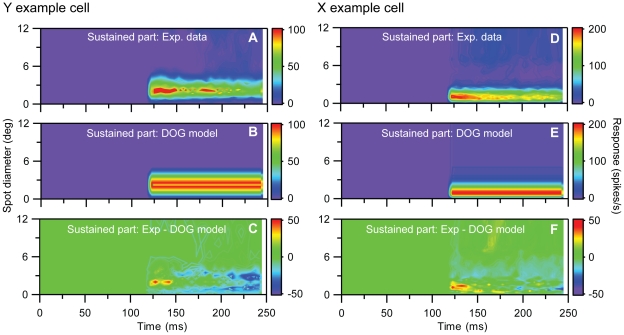
Fits to spatial part of sustained component of TS model for example on-center Y and X neurons in **Figs. 1** and **2**. **A**, Last part (t>125 ms) of experimental Y-neuron response data used in fit. **B**, Best fit to DOG model in Eq. (10) representing the spatial part of the sustained component in the TS-model. **C**, Deviation between experimental results (A) and model results in (B). Error *ε* (cf. Eq. 1) is 0.053. **D–F**, Same as (A)–(C) for the X-neuron. The deviation between experimental results (D) and model results (E) corresponds to an error *ε* = 0.019. The fitted parameter values (*A_s_*, *B_s_*, *a_s_*, *b_s_*) from both fits are listed in Table 1.

We first focus on the *sustained* component: A principal components analysis of the last part of the sustained response (t>122.5 ms) for our example Y- and X-neurons revealed that the first principal component in both cases could account for more than 99.4% of the data, i.e., the ‘error’ was less than 0.6% (results not shown). This suggests that a spatiotemporally separable function *R_s_*(*t,d*)* = F_s_*(*t*)*G_s_*(*d*) can account well for this part of the response, and we further found that the spatial part *G_s_*(*d*) could be excellently modeled as a DOG (Eq. 10). The detailed spatial shape of this sustained DOG was found by fitting the DOG-model area summation curve to all sustained data (t>122.5 ms), and the results for the fits to the example Y- and X-neuron data are shown in Fig. 8. The temporal profile of the sustained part of the response *F_s_*(*t*) was modeled as a (low-pass) rising exponential function (Eq. 13), but the fitting of the temporal parameters *τ_s_* was done in the final optimization routine involving the complete TS model. The onset time of the sustained component *t_s_* was fixed to 62.5 ms in this final optimization, in accordance with the results found in the Experimental Analyses section above.

**Figure 9:**
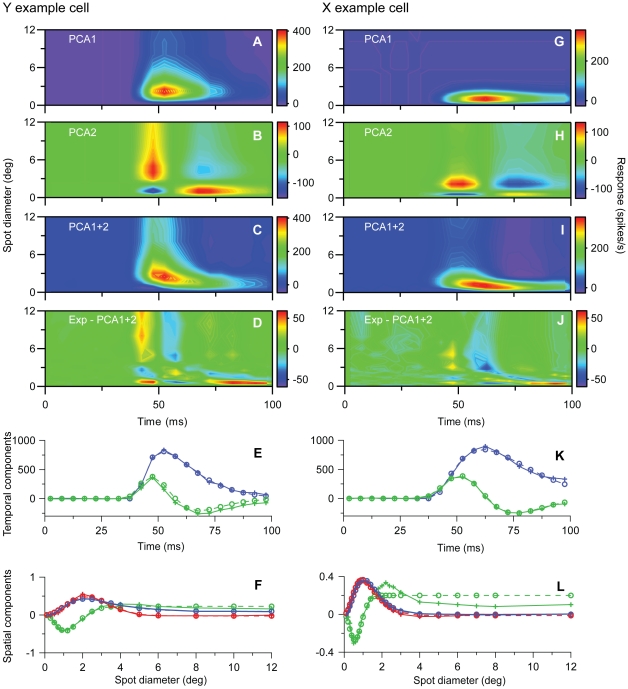
Principal components analysis (PCA) of early part of response data (t<100 ms) for example on-center Y and X neurons in **Figs. 1** and **2**. **A**,**B**, 1^st^ and 2^nd^ principal components, respectively, for the Y-neuron response data, i.e., contributions from terms with *n = 1* and *n = 2* in Eq. (3). **C**, Sum of contributions from two first principal components (and background activity) for Y neuron. **D**, Deviation between experimental results for Y neuron and PCA results in (C). Error *ε* (cf. Eq. 1) is 0.044. **E**, Fitted transient temporal function *F_t1_*(*t*) (Eq. 11, blue dashed line) to 1^st^ temporal PCA component (blue solid line), and fitted transient temporal function *F_t2_*(*t*) (Eq. 12, green dashed line) to 2^nd^ temporal PCA component (green solid line) for early part ( t<97.5 ms) of Y-neuron data. **F**, Blue dashed line: Fitted DOG spatial functions (Eq. 10) to 1^st^ spatial PCA component of early part (t<97.5 ms) of Y-neuron data (blue solid line). Green dashed line: Corresponding DOG function fit to the 2^nd^ spatial PCA component (green solid line). The best fit of a DOG function (red dashed line) to the 1^st^ spatial PCA component of the *last* part of the Y-neuron data is also shown (red line). **G–L**, Same as (A)–(F) for X-neuron response data. The deviation between experimental results and PCA results (I) corresponds to an error *ε* = 0.021.

We next focus on the *transient* part: a PCA analysis of the first part (t<97.5 ms) of the response for the example Y- and X-neurons revealed that the first principal components in both cases were found to account for less than 90% of the data, see Fig. 9. This demonstrates that a simple spatiotemporally response function *R_t_*(*t,d*)* = F_t_*(*t*)*G_t_*(*d*) will be insufficient. The PCA analysis further revealed that the first and second principal components combined in both cases accounted for more than 97.5% of the stimulus-evoked data, and that these two principal components together are sufficient to capture the temporal shrinking of the RF-center size (Fig. 9C and 9I). We thus chose to model the transient part of the response by a sum over two spatiotemporally separable functions, i.e., *R_t_*(*t,d*) = *F_t1_*(*t*)*G_t1_*(*d*)+*F_t2_*(*t*)*G_t2_*(*d*).

To choose the functional forms of *F_t1_*(*t*), *F_t2_*(*t*), *G_t1_*(*d*), and *G_t2_*(d) we investigated the temporal scores (*f_n_*(*t_i_*)) and spatial loadings (*g_n_*(*d_i_*)) of these first two principal components of the transient response. The temporal scores of the first PCA components were found to have monophasic time courses (Figs. 9E and 9K), and we chose to model *F_t1_*(*t*) using the monophasic function in Eq. (11). This function were found to be able describe the temporal scores of the first principal components excellently (Figs. 9E and 9K). The second PCA components were found to have biphasic time courses, and we thus chose to model it using the function *F_t2_*(*t*) in Eq. (12), which essentially is the time-derivative of *F_t1_*(*t*). *F_t2_*(*t*) were found to fit the temporal scores of the second principal component for both example cells very well (Figs. 9E and 9K). The spatial loads of both the first and second principal components were found to be well accounted for by the DOG function except for one feature: the spatial load of the second PCA component has two extremal points for the example X cell, a feature that cannot be captured by the DOG model (Figs. 9F and 9L). We thus chose to model also the spatial components of the transient parts *G_t1_*(*d*) and *G_t2_*(d) as DOG response functions (Eq. 10).

**Figure 10:**
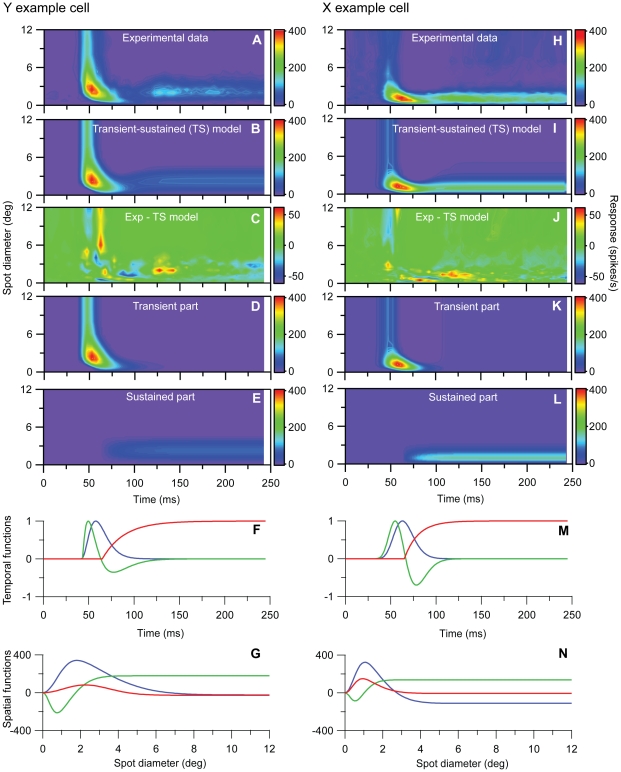
Fits to transient-sustained (TS) model for example on-center Y and X neurons in **Figs. 1** and **2**. **A**, Experimental Y-neuron response data. **B**, Best fit to TS model in Eq. (14). **C**, Deviation between experimental results (A) and model results in (B). Error *ε* (cf. Eq. 1) is 0.029. **D**, Transient component only, i.e., *R*(*t,d*) = [R*_bkg_+F_t1_*(*t*)*G_t1_*(*d*)+*F_t2_(t*)*G_t2_*(*d*))]_+_. **E**, Sustained component only, i.e., *R*(*t,d*) = [R*_bkg_+F_s_*(*t*)*G_s_*(*d*)]_+_. **F**, Fitted transient temporal functions *F_t1_*(*t*) (Eq. 11,blue line) and *F_t2_*(*t*) (Eq. 12, green line), and sustained temporal function *F_s_*(*t*) (Eq. 13, red line) for Y-neuron. **G**, Fitted transient spatial functions *G_t1_*(*d*) (blue line) and *G_t2_*(*d*) (green line), and sustained spatial function *G_s_*(*d*) (red line) for Y-neuron. All spatial functions are modeled as DOGs, cf. Eq. (10). **H–N**, Same as (A)–(G) for the X-neuron response data. The deviation between experimental results (H) and model results (I) corresponds to an error *ε* = 0.022. The fitted parameter values from both fits are listed in Table 1.

The fits of our full TS-model in Eq. (14) to the example Y and X cell response data are shown in Figs. 10B and 10I. The fitting errors are only 2.9% and 2.2%, respectively, and importantly we see that the TS-model can account for the shrinking of the RF for early times. In Figs. 10D, 10E, 10K, and 10L we illustrate how the individual transient and sustained components contribute to the total response function. Figs. 10F, 10G, 10M, and 10N show the corresponding fitted temporal (*F_t1_*(*t*), *F_t2_*(*t*), *F_s_*(*t*)) and spatial functions (*G_t1_*(*d*), *G_t2_*(*d*), *G_s_*(*d*)) constituting the building blocks of *R_TS_*(*d*). It is notable that the spatiotemporal characteristics of the first and second parts of the transient response are very different: while the first component has the traditional shape with a monophasic temporal function and an area summation curve corresponding to a center smaller than the surround, the second component has a biphasic temporal function and an unconventional area summation curve that has a first negative peak for spot diameters much smaller than the RF-center size and then changes sign for larger spot diameters. Whether the separation into these two components of the transient response relates in any way to different physiological mechanisms is, however, unclear. The resulting fitted parameters for the example Y- and X-neurons are listed in Table 1.

**Table 1:** Best-fit parameters from fitting the transient-sustained (TS) model in Eq. (14) to response data for example Y and X cells, cf. Fig. 10.

	Y cell	X cell
*t_1_* (ms)	38.7	19.1
*τ_1_* (ms)	5.5	2.7
*n_1_*	3.0	15.0
*A_1_* (spikes/s)	477	527
*B_1_* (spikes/s)	500	637
*a_1_* (deg)	0.56	0.35
*b_1_* (deg)	1.91	1.04
*t_2_* (ms)	40.7	20.2
*τ_2_* (ms)	9.7	3.1
*n_2_*	2.1	14.0
*A_2_* (spikes/s)	537	345
*B_2_* (spikes/s)	358	207
*a_2_* (deg)	0.83	0.53
*b_2_* (deg)	0.26	0.22
*τ_s_* (ms)	24.0	15.2
*A_s_* (spikes/s)	174413	226
*B_s_* (spikes/s)	174440	232
*a_s_* (deg)	1.186	0.30
*b_s_* (deg)	1.187	0.89
*R_bkg_* (spikes/s)	6.5^*^	15.3^*^
*t_s_* (ms)	62.5^*^	62.5^*^
error (*ε*) TS-model	0.029	0.022
error (*ε*) CS-model	0.071	0.040

The number marked with asterisks are not fitted: *R_bkg_* is found by averaging the background response prior to the stimulus-evoked response, and *t_s_* is fixed at 62.5 ms (see main text). Fitting errors (Eq. 1) for both the TS-model and the CS-model (center-surround model, Eq. 6) are also listed.

**Figure 11:**
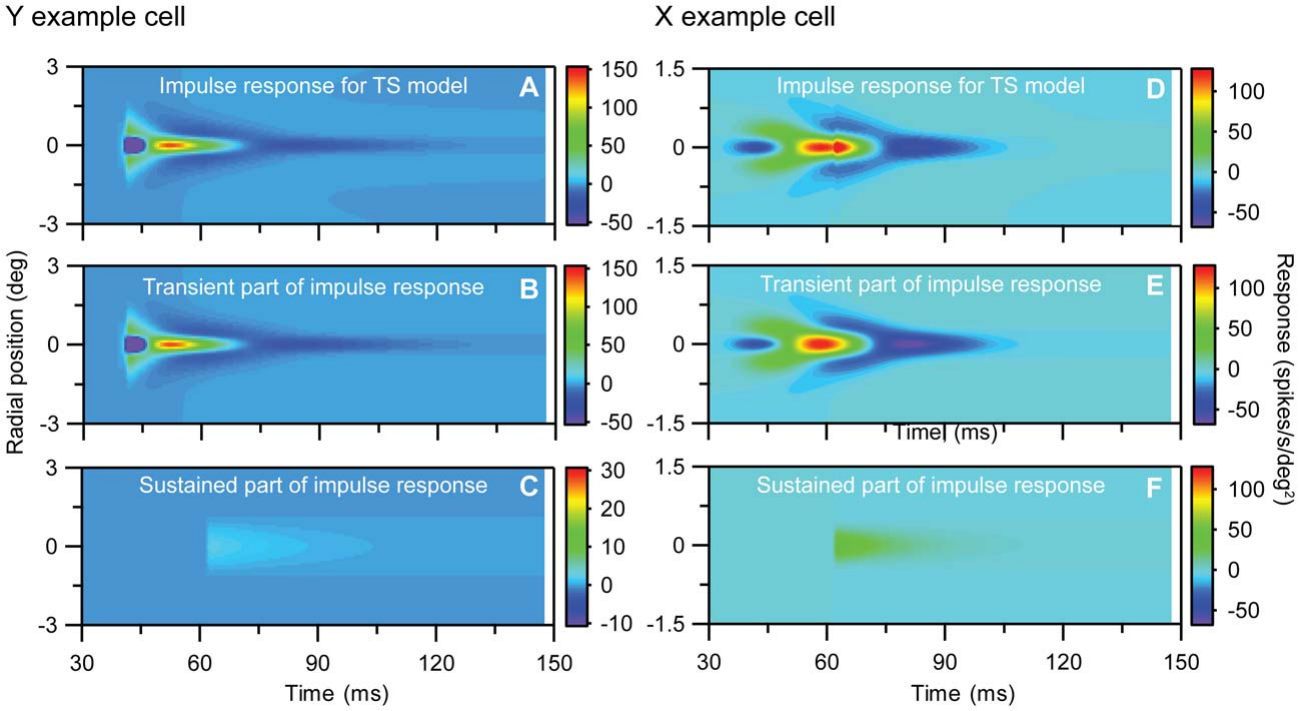
Predicted spatiotemporal impulse-response function *D_TS_*(*t,r*), cf. **Eq.(16)**, for the transient-sustained (TS) model for example on-center Y and X neurons in **Figs. 1** and **2**. All model parameters correspond to the fit depicted in Fig. 10 and are listed in Table 1. **A.** Predicted impulse-response function for full TS-model for Y neuron. **B.** Contribution from transient part (*f_t1_*(*t*) *g_t1_*(*r*)+*f_t2_*(*t*) *g_t2_*(*r*)). **C.** Contribution from sustained part (*f_s_*(*t*) *g_s_*(*r*)). **D**–**F.** Same as (A)–(C) for the X-neuron. Notice that (i) the color scale in C and F differ from the scale in the other corresponding color maps and (ii) that the negative response for the Y-neuron has been truncated at the value −50 spikes/s/deg^2^ in panels A and B.

With all model parameters determined by fitting the TS-model to our spot-response data, we can now calculate a corresponding spatiotemporal impulse-response function *D_TS_(r)*, cf. Eq. (16), which predicts the spatiotemporal firing-rate response to tiny test spots on for only a tiny period of time. Such a mapping from a measured response with one stimulus to predicting the response for another stimulus, requires the system to be linear, an assumption that appears particularly questionable for Y cells [37]. Regardless, in Figs. 11A and 11D we show the total impulse response predicted by Eq. (16). Figs. 11B and 11E show the contribution from the transient part, and Figs. 11C and 11F the contribution from the sustained part. The figures further illustrate that for the example Y cell the sustained part is much weaker than the transient, while the difference is smaller for the example X cell.

**Figure 12:**
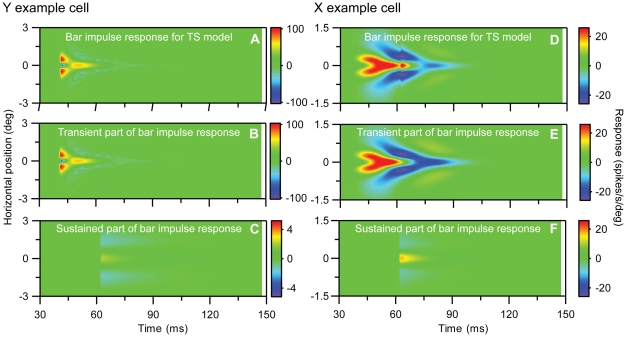
Predicted ‘one-dimensional impulse response’, i.e., impulse response for long and thin bars, for the transient-sustained (TS) model for example on-center Y and X neurons in **Figs. 1** and **2**. This impulse-response function of the form given in Eq. (16), but with the spatial functions *g_m_*(*r*) replaced by the function *g_bar,m_*(*x*) listed in Eq. (21). The test bar in the example has a length *L* = 10 deg. All model parameters correspond to the fit depicted in Fig. 10 and are listed in Table 1. **A.** Predicted receptive-field function for full TS-model for Y neuron. **B.** Contribution from transient part (*f_t1_*(*t*) *g_bar,t1_*(*x*)+*f_t2_*(*t*) *g_bar,t2_*(*x*)). **C.** Contribution from sustained part (*f_s_*(*t*) *g_bar,s_*(*x*)). **D**–**F.** **S**ame as (A)–(C) for the X-neuron. Notice that (i) the color scale in C and F differ from the scale in the other corresponding color maps and (ii) that the negative response for the Y-neuron has been truncated at the numerical value −100 spikes/s/deg in panels A and B.

**Figure 13:**
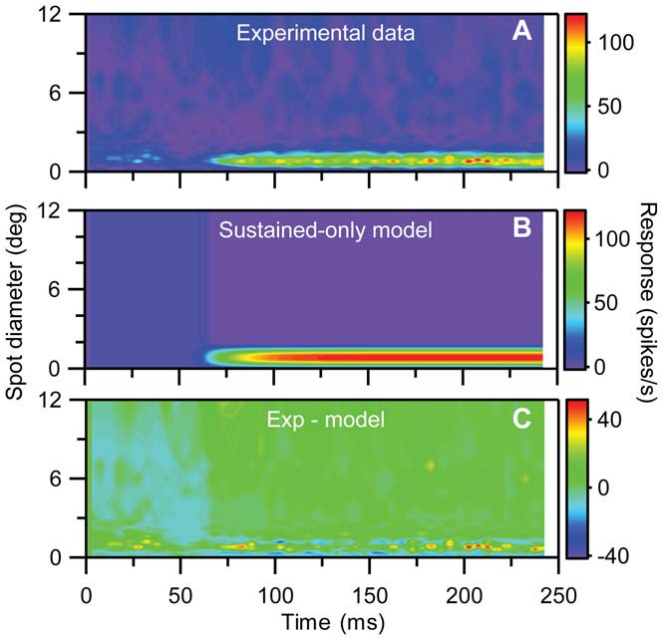
Fit of sustained-only model to the response data of the lagged cell in **Fig. 4**. **A**, Experimental data. **B**, Best fit to sustained-only model in Eq. (15). **C**, Deviation between experimental results (A) and model results in (B). Error *ε* (cf. Eq. 1) is 0.086.

LGN cells have also been studied using reverse-correlation techniques where randomized long bar stimuli have been used instead of small test spots [14], [15]. The resulting ‘one-dimensional’ impulse-response function is also straighforwardly predicted for our TS-model, cf. Eq. (21) in Methods, again with the caveat that linearity must be assumed. In Fig. 12 we show for completeness these predicted one-dimensional impulse responses for our example cells. Finally we also found the sustained-only model in Eq. (15) to account well for the experimental data for lagged neurons. The best fit to the example X-off lagged neuron in Fig. 4 is shown in Fig. 13.

## Discussion

The experimental results demonstrated an initial pronounced transient shrinkage of the RF-center and a subsequent more stable center-width during the static spot stimulation for all non-lagged neurons, consistent with our previous results [16]. The color-map images of the responses and the plots of spot-width vs. time indicated a discontinuity in the change of RF-center width rather than a continuous change at the transition from the first to the second component. This was substantiated by results from the detailed analyses of the spatial summation curves, which showed an inflection or bimodal shape of the curves in the range of transition between the two components, and indicated a partial temporal overlap between the two components. These results suggest that the transient and the sustained part reflect contributions from two distinctly different neuronal mechanisms that operate in parallel with partial temporal overlap. Spatially, both mechanisms have antagonistic center-surround organization as demonstrated by the summation curves. Thus, rather than simply reflecting a continuous change of balance between an excitatory center and a delayed inhibitory surround (e.g. [15]), the dynamics of the RF-center width seems to involve two distinctly different sets of spatiotemporal mechanisms.

It could be argued that the initial changes in the color-map images reflect primarily temporal response properties rather than spatial changes in the RF, i.e. that the response to larger spots have shorter latency than the response to smaller spots simply due to differences of spatial summation. However, the short duration of the response to the large spots is inconsistent with this hypothesis. Moreover, we previously [16] demonstrated that a small eccentric stimulus spot presented outside the minimum RFC but inside the maximum RFC elicited only a fast and transient response consistent with a real shrinkage of the RFC.

The results from the mathematical modeling support the conclusions from the experimental data. In the modeling, we systematically investigated various models for the spatiotemporal response and compared them with our detailed time-resolved data. The *principal components analysis (PCA)* clearly demonstrated that a model response function for non-lagged neurons must at least include a sum over two different spatiotemporal functions. One such type of candidate model is the commonly assumed *center-surround (CS) models* [12], [14], [15], [24] built up as a sum of a center term and a surround term, and a fixed time lag between the two components. However, our mathematical analysis clearly showed that the CS-model was unable to capture the salient features of the spatiotemporal response, in particular the shrinkage of the RF-center during the transient phase. This conclusion not only applied to the version of the CS-model with the particular choices of the temporal weight functions assumed in, e.g., Cai et al. [14] and Allen and Freeman [15]; our analysis with a non-parametric fit of the CS-model, corresponding to allowing 100 model parameters to vary in the fit, also gave a poor fit. Our conclusion from this analysis was thus that no CS-model could account for the present data, and we therefore investigated alternative mathematical models.

Fitting of the data to the DOG model for each time slice separately supported the conclusion from the direct analysis of the experimental data, namely that the data are most naturally represented by a sum of an early transient component and a partially overlapping sustained component. Further mathematical analysis revealed that two spatiotemporal components are needed to represent the transient part of the response with a time-dependent RF-center size, while a single component is sufficient for the sustained component. Our new *transient-sustained (TS) model*, described by a sum of three spatiotemporal components, accounted excellently for the experimental data. The successful fit to the TS-model involved 19 freely varying model parameters rather than the 100 model parameters of the unsuccessful non-parametric fits to the CS-model. Accordingly, the better fit of the TS-model came despite of a much smaller number of fitting parameters. Use of the Akaike information criterion [38], which penalizes models with many fitting parameters, would in fact favor the TS-model even more compared to the CS model. The crucial new feature of the TS-model is the assumption of the response being given as a sum over a transient and a sustained component. To effectively capture the rapid shrinking of the receptive-field center size for the transient component, we chose to model this component as a sum over two product functions mimicking the two first PCA components of the transient part of the response. This choice is mathematically convenient, but it is unclear if, or to what extent, this decomposition of the transient component relates to two different underlying physiological mechanisms.

In the human visual system the existence of spatiotemporally distinct transient and sustained channels were suggested by a number of early psychophysical studies (e.g. [1], [39]–[44]). The transient channels operate at low and moderate spatial frequencies mediating brief response (∼100 ms, [42]) at onset or offset of a flashed stimulus, the sustained channels operate at high spatial frequencies mediating response for the whole duration of the stimulus. The two channels have been related respectively to Y (transient) and X (sustained) retinal ganglion cells and dLGN neurons (e.g. [39]–[45]), but this link seems less likely since both Y and X retinal ganglion cells and nonlagged dLGN neurons typically respond to a flashed stimulus with an initial transient followed by a sustained response. However, the dynamics of RF-organization we found are consistent with the reinterpretation that the two psychophysically defined channels may actually reflect two different components in the receptive field evolvement in both Y and X neurons. As illustrated by Figs. 1A, 2A and 3A, the response to large spots (low spatial frequencies) was limited to the initial response and accordingly transient like responses in the psychophysically defined ‘transient channel’. Gradually during the time sequence, the response becomes limited to smaller spots, and the response becomes more sustained like the psychophysically defined ‘sustained channel’. This relationship would suggest that a similar dynamics of RF-center size also exists in the human visual system.

Lagged neurons are generated in dLGN by transformation of the characteristic transient-sustained response pattern of retinal ganglion cells into the delayed and sustained response pattern of the lagged neurons [19]. The transformation is presumably caused by fast intrageniculate feed-forward inhibition that eliminates the initial transient response component since direct application of GABA-A receptor antagonists on a lagged neuron changes its response into a nonlagged pattern [36]. It is of interest in this connection that our modeling demonstrated that the spatiotemporal characteristics of the lagged neurons were adequately accounted for by the sustained-only model (Eq. 15).

The underlying neuronal mechanisms for the dynamics of the RF-center width of the nonlagged neurons are unknown. We previously demonstrated that the retinal input to nonlagged dLGN neurons has a similar dynamics of RF-center width during spot stimulation as the dLGN neurons [16], indicating that the initial pronounced shrinkage of the RF-center must at least mainly be of retinal origin. It is of interest in this connection that Passaglia et al. [46] showed increased firing in some X and most Y retinal ganglion cells to stimulation with gratings of low spatial but high temporal frequency outside the classical RF. This is consistent with the low spatial resolution we found in the initial response of the retinal input to nonlagged neurons in dLGN [16]. However, the retinal mechanisms that generate the key characteristics of the transient component are unclear. The initial very wide RF-centers might reflect lateral spread of excitation between retinal neurons through neuronal gap junctions [47], for instance already between photoreceptors [48]–[51], beside the convergence of synaptic input in the vertical retinal pathway. Possible mechanisms for the fast constriction of the RF-center during rapidly increasing center-surround antagonism could be increasing lateral summation of activity across horizontal cells in the outer plexiform layer or interactions in the inner plexiform layer, for instance interaction between wide-field and transient amacrine cells (e.g. [52], [53]) and bipolar cells.

It is generally assumed that the width of the RF-center of a neuron is directly related to its spatial resolution for details in visual stimulus patterns. Accordingly, the change of center width during the visual stimulation strongly suggests that this dynamics has an important role in the coarse-to-fine processing manifested in several phenomena of visual perception (cf. e.g. [1], [2]). In particular, the transient and sustained component of the response may have different functional roles. The fast onset, high peak firing rate, and coarse spatial resolution of the transient response component is well suited for functions related to object and pattern detection [54], [55], whereas the subsequent sustained response component with its higher spatial resolution is well suited for functions related to fine discrimination and detailed pattern analyses. Moreover, it is reasonable to suggest that the dynamics of several types of response selectivity observed in visual cortex is largely a reflection of the dynamics of geniculate input to the cortical circuits that generate the various types of stimulus selectivity. This includes dynamics of spatial frequency selectivity [4], [6], increasing sharpness of disparity tuning [5], [56], orientation discriminability or selectivity [3], [57], [58], shape selectivity [7], or shrinkage of cortical RF-subregions [9]. Interestingly, this response dynamics seems to be mainly of retinal origin [16].
